# High prevalence of exposure to the child welfare system among street-involved youth in a Canadian setting: implications for policy and practice

**DOI:** 10.1186/1471-2458-14-197

**Published:** 2014-02-24

**Authors:** Brittany Barker, Thomas Kerr, Gerald Taiaiake Alfred, Michelle Fortin, Paul Nguyen, Evan Wood, Kora DeBeck

**Affiliations:** 1 British Columbia Centre for Excellence in HIV/AIDS, St. Paul's Hospital, 608-1081 Burrard Street, Vancouver BC V6Z 1Y6, Canada; 2 School of Public Policy, Simon Fraser University, Vancouver, Canada; 3 Department of Medicine, University of British Columbia, Vancouver, Canada; 4 Indigenous Governance, University of Victoria, Victoria, Canada; 5 Watari Counselling & Support Services Society, Vancouver, BC, Canada

**Keywords:** Child welfare system, Foster care, Government care, Street-involved youth, Youth substance use

## Abstract

**Background:**

Street-involved youth are more likely to experience trauma and adverse events in childhood; however, little is known about exposure to the child welfare system among this vulnerable population. This study sought to examine the prevalence and correlates of being in government care among street-involved youth in Vancouver, Canada.

**Methods:**

From September 2005 to November 2012, data were collected from the At-Risk Youth Study, a prospective cohort of street-involved youth aged 14–26 who use illicit drugs. Logistic regression analysis was employed to identify factors associated with a history of being in government care.

**Results:**

Among our sample of 937 street-involved youth, 455 (49%) reported being in government care at some point in their childhood. In a multivariate analysis, Aboriginal ancestry (adjusted odds ratio [AOR] = 2.07; 95% confidence interval [CI]: 1.50 – 2.85), younger age at first “hard” substance use (AOR = 1.10; 95% CI: 1.05 – 1.16), high school incompletion (AOR = 1.40; 95% CI: 1.00 – 1.95), having a parent that drank heavily or used illicit drugs (AOR = 1.48; 95% CI: 1.09 – 2.01), and experiencing physical abuse (AOR = 1.90; 95% CI: 1.22 – 2.96) were independently associated with exposure to the child welfare system.

**Conclusions:**

Youth with a history of being in government care appear to be at high-risk of adverse illicit substance-related behaviours. Evidence-based interventions are required to better support vulnerable children and youth with histories of being in the child welfare system, and prevent problematic substance use and street-involvement among this population.

## Background

Children who are taken into government care, such as orphanages, foster homes, group homes, or otherwise become a ward of the state, constitute a highly vulnerable population. Although the transition into government care is intended to provide a safer and more stable environment, data indicates that many youth continue to struggle emotionally, physically, academically, and behaviourally during and after care [1-7]. In the Canadian province of British Columbia, the ministry responsible for child welfare (Ministry of Children and Family Development) estimated that in 2010/2011, 49.9% of all youth in continuing government care were in special education classes and 63.8% were of Aboriginal ancestry [8]. These youth were also more likely to have intensive behavioural problems, serious mental illness, physical disabilities, and chronic health impairments [8].

The longer-term trajectories for children and youth who are taken into government care further underscores the vulnerability of this population. The struggle for youth exposed to the child welfare system continues well into early adulthood with elevated rates of poverty, underemployment, housing instability, incarceration, unplanned pregnancies and subsequent government involvement with parenting, mental health, physical health and substance use issues [5-7,9-13]. Given this profile, youth with a history of being in government care may be at greater risk for substance misuse and street-involvement. As such, we sought to examine the prevalence and correlates of youth with a history of being in government care among a cohort of street-involved youth who use illicit drugs in Vancouver, Canada.

## Methods

Data for this cross-sectional study was collected at baseline study visits between September 2005 and November 2012, from the At-Risk Youth Study (ARYS), a prospective cohort of street-involved youth in Vancouver, Canada. Youth were eligible if they were between the ages of 14–26 at time of enrolment, had used illicit “hard” drugs in the past 30 days (e.g. crack, cocaine, heroin, or crystal methamphetamine) and provided written informed consent. The study has been described in detail elsewhere [14]; however, in brief, recruitment was undertaken using a snowball sampling approach with extensive outreach efforts in order to maximise the representativeness of this sample. In short, at baseline and subsequent semi-annual follow-up interviews, participants complete an interviewer-administered questionnaire and provide blood samples for HIV and HCV serology. The survey includes items on sociodemographic information, substance use patterns, sexual and drug-related risk behaviours, interactions with the criminal justice system, and engagement with health and social services. At each study visit, participants receive a $20 monetary compensation (Canadian dollars) and the research ethics board of the University of British Columbia has approved the study.

The main outcome examined in the current analysis was a history of being in government care. This was defined as responding “yes” to the question: “As a child, did you ever live in an orphanage, a foster home, a group home, as a ward of the state, or away from your parents for a month or more (not including vacations)?” The comparison group was youth who reported not having been exposed to the child welfare system.

Explanatory variables thought to be potentially associated with our outcome of interest based on prior studies examining transitions in and out of government care [12,13,15-17], including the following sociodemographic data: gender (female vs. male); Aboriginal ancestry (self-identified as First Nations, Inuit, Métis vs. other); high school incompletion (yes vs. no); having a parent that drank heavily or used illicit drugs during their childhood (yes. vs. no); homelessness, defined as having no fixed address, sleeping on the street, couch surfing, or staying in a shelter or hostel in the last six months (yes vs. no); and living in the Downtown Eastside (DTES) in the last six months, defined as living in Vancouver’s substance use epicenter and poorest neighborhood (yes vs. no). Behavioural and substance use variables, based on activities in the last six months, included: injection substance use (yes vs. no); drug overdose (yes vs. no) defined as having an adverse reaction as a result of consuming too much of a drug; daily injection or non-injection heroin use (yes vs. no), defined as any daily heroin use, regardless of the mode of administration (e.g., smoked, snorted, injected, swallowed, etc.); daily injection or non-injection cocaine use (yes vs. no); daily crack cocaine smoking (yes vs. no); daily injection or non-injection crystal methamphetamine use (yes vs. no); syringe sharing, defined as borrowing or lending used syringes (yes vs. no); engaging in sex work defined as exchanging sex for money, shelter, drugs or other commodities (yes vs. no); and participation in drug dealing (yes vs. no). Other factors included: age at first hard substance use, defined as the age participants first used non-injection crack, cocaine, heroin, or crystal methamphetamine (per year younger); testing positive for Hepatitis C virus (yes vs. no); incarceration, defined as being in detention, prison, or jail overnight or longer in the previous six months (yes vs. no); having ever been the victim of sexual abuse (yes vs. no); having ever been the victim of physical abuse (yes vs. no); and recently experiencing an act of violence, defined as being attacked, assaulted, or suffering violence in the previous six months (yes vs. no).

In the bivariate analysis, dichotomous variables were analysed using Pearson’s chi-square test and continuous variables were analysed using the Mann–Whitney test. To evaluate factors independently associated with our outcome of interest, all variables with p-values that were *p* < 0.1 in bivariate analyses were considered in a multivariate logistic regression. A backward model selection procedure was used to identify the multivariate model with the best overall fit, as indicated by the lowest Akaike Information Criterion [AIC] value [18]. All statistical analyses were performed using SAS software version 9.3 [SAS, Cary, NC]. All p-values are two sided.

## Results

Over our study period, 937 youths enrolled in the ARYS cohort who provided complete responses to the questionnaire items were included in this analysis. In total, 91 (8.6%) participants were excluded from the original sample of 1028 for not providing complete answers to the questions included in the analysis. Among this sample, 292 (31%) were female, 224 (24%) were of Aboriginal ancestry, and the median age was 21.0 (interquartile range [IQR]: 20.0 - 23.0). In total, 455 (49%) participants reported being in government care at some point in their childhood.

The characteristics of the study sample stratified by having a history of being in government care in childhood, along with the bivariate and multivariate analyses of factors associated with childhood exposure to government care are presented in Table 1. In multivariate logistic regression factors that remained independently associated with our outcome of interest included: Aboriginal ancestry (adjusted odds ratio [AOR]: 2.07, 95% confidence interval [CI]: 1.50 – 2.85), younger age at first hard substance use (AOR: 1.10, 95% CI: 1.05 – 1.16), high school incompletion (AOR: 1.40, 95% CI: 1.00 – 1.95), having a parent that drank heavily or used illicit drugs (AOR: 1.48, 95% CI: 1.09 – 2.01), and being a victim of physical abuse (AOR: 1.90, 95% CI: 1.22 – 2.96).

**Table 1:** Baseline, bivariate and multivariable analyses of factors associated with a history of being in government care among street-involved youth in Vancouver, Canada (n = 937)

	**Government care exposure**					
**Characteristic**	**Yes**	**No**	**Odds ratio, (95% CI**^**a**^**)**	***p-*** **value**	**Adjusted odds ratio, (95% CI**^**a**^**)**	***p-*** **value**
	***n*** **= 455, *n *** **(%)**	***n*** **= 482 *n *** **(%)**				
**Age at first substance use**	15^**b**^	16^**b**^	1.12 (1.06 – 1.18)	<0.001	1.10 (1.05 – 1.16)	<0.001
Per year younger	(13–16)^**c**^	(14–17)^**c**^				
**Gender**						
Female vs. Male	157 (35)	135 (28)	1.35 (1.03 – 1.79)	0.032		
**Aboriginal ancestry**						
Yes vs. No	139 (31)	85 (18)	2.05 (1.51 – 2.80)	<0.001	2.07 (1.50 – 2.85)	<0.001
**High school incompletion**						
Yes vs. No	375 (82)	346 (72)	1.84 (1.35 – 2.52)	<0.001	1.40 (1.00 – 1.95)	0.049
**Parental alcohol/ Substance use**						
Yes vs. No	354 (78)	320 (66)	1.77 (1.33 – 2.37)	<0.001	1.48 (1.09 – 2.01)	0.012
**Lives in the DTES**^**d**^						
Yes vs. No	124 (27)	139 (29)	0.92 (0.70 – 1.23)	0.590		
**Homeless**^**d**^						
Yes vs. No	349 (77)	352 (73)	1.22 (0.90 – 1.64)	0.200		
**Injection substance use**^**d**^						
Yes vs. No	132 (29)	150 (31)	0.91 (0.68 - 1.20)	0.482		
**Daily heroin use**^**d, e**^						
Yes vs. No	49 (11)	68 (14)	0.74 (0.50 – 1.09)	0.123		
**Daily cocaine use**^**e**^						
Yes vs. No	19 (4)	21 (4)	0.96 (0.51 – 1.80)	0.891		
**Daily crack use**^**d**^						
Yes vs. No	85 (19)	87 (18)	1.04 (0.75 – 1.45)	0.803		
**Daily crystal meth use**^**e**^						
Yes vs. No	72 (16)	54 (11)	1.49 (1.02 – 2.18)	0.039		
**Drug overdose**^**d**^						
Yes vs. No	58 (13)	46 (10)	1.39 (0.92 – 2.09)	0.120		
**Hepatitis C positive**						
Yes vs. No	90 (20)	64 (13)	1.61 (1.14 – 2.29)	0.008	1.36 (0.94 – 1.97)	0.099
**Syringe sharing**^**d**^						
Yes vs. No	38 (8)	37 (8)	1.10 (0.68 – 1.76)	0.703		
**Victim of violence**^**d**^						
Yes vs. No	214 (47)	210 (44)	1.15 (0.89 – 1.49)	0.287		
**Incarcerated**^**d**^						
Yes vs. No	96 (21)	86 (18)	1.23 (0.89 – 1.70)	0.208		
**Physical abuse**						
Yes vs. No	413 (91)	397 (82)	2.11 (1.42 – 3.12)	<0.001	1.90 (1.22 – 2.96)	0.005
**Sexual abuse**						
Yes vs. No	345 (76)	317 (66)	1.63 (1.23 – 2.17)	<0.001	1.29 (0.93 – 1.78)	0.124
**Sex work**^**d**^						
Yes vs. No	54 (12)	43 (9)	1.38 (0.90 – 2.10)	0.140		
**Drug dealing**^**d**^						
Yes vs. No	254 (56)	255 (53)	1.13 (0.87 – 1.46)	0.370		

Note: ^a^CI = Confidence Interval; ^b^Median; ^c^Interquartile Range; ^d^Refers to activities in the past six months; ^e^Injection and Non-injection.

## Discussion

In the present study of street-involved youth in Vancouver, Canada, we found that experiences of being in government care in childhood were common, especially for youth who were of Aboriginal ancestry, victims of physical abuse, had not completed high school, had parents that drank heavily or used illicit substances, and had initiated first hard substance use at an earlier age. In Canada roughly 0.3% of youth have been exposed to the child welfare system [19], suggesting that street-involved youth who use substances are over 160 times more likely to have been in government care compared to the general population of youth.

Some of our findings are consistent with previous research. For example, youth exposed to the child welfare system have been found to be more likely to engage in risky behaviours and substance use later in life versus their peer group [1,7,13]. One US based study found higher intensity substance use patterns among homeless youth with a history of care compared to homeless youth without a history of care [9]. Similarly, our study found that youth exposed to child welfare were significantly more likely to initiate hard substance use at an earlier age. While we did not detect any other differences in substance use patterns among those with a history of being in government care, this may be attributed in part to the composition of our sample, which is restricted to high-risk youth that already engage in hard substance use. While it is hard to delineate whether the events in a youth’s life preceding government intervention, or, the experiences during their tenure in care have the greatest influence and impact on risk of illicit substance use and becoming street-involved later in life, it is evident that more interventions are necessary to support these children and youth in avoiding substance use and street-involvement.

Youth in our sample who were formerly in government care were twice as likely to be of Aboriginal ancestry, which is consistent with government data [8,19]. The Aboriginal youth population represents approximately five percent of the total youth population in Canada; however, they account for approximately 50% of the children and youth in government care [19]. Previous research has identified the overrepresentation of ethnic minorities in child welfare services across high income nations [20-24]. Community organisations and researchers estimate there are three times as many Aboriginal children in the custody of the Canadian government today than there were during the time of residential schools – the government-funded, Church administered network of culturally destructive and harmful boarding schools that illegally removed thousands of Aboriginal children from their homes predominantly from the late 1800s to the 1930s [21,23,25]. The majority of interventions in Aboriginal child welfare are due to charges of neglect, often in the context of perpetual poverty, inadequate housing, food insecurity, parental substance use, and other remnants of colonisation which continue to exacerbate the difficulties many Aboriginal parents face [25]. These and the intergenerational trauma experienced by families and communities as a result of losing more than a generation’s example of parental-modeling has been associated with the high prevalence of neglect charges [21,25-27].

Interventions in Aboriginal child welfare should be driven by the respective community they affect, with First Nations and Aboriginal communities directing solutions that build-on their unique strengths and cultures. The proliferation of on-and-off reserve Aboriginal child welfare organisations in recent years has demonstrated some success in keeping children and youth tied to their communities and culture [28]. Early research indicates that Aboriginal children experience fewer emotional problems when care arrangements keep them tied to their culture and community [29,30]. Indigenous scholars and activists have called for the relationship between community development and child wellbeing to be examined, while continuing to increase culturally-based programs focused on prevention rather than intervention [21,25,26,30,31]. In addition, strengthening kinship care by providing appropriate and equitable support to Aboriginal relatives who assume custody of youth in care would likely help these youth remain tied to their communities and culture. Being cared for by relatives has been shown to provide an element of stability and improved outcomes for youth, although typically little support and funding from governments is provided to kinship caregivers, making it difficult for relatives to take on the financial burden of raising a youth [32-35]. Our study findings indicate that continued attention and efforts to improve outcomes for vulnerable Aboriginal youth and to address their overrepresentation in the child welfare system are required.

Our study found that having a parent that drank heavily or used illicit substances was associated with a history of being in government care. Previous research has demonstrated a relationship between parental substance use and subsequent child welfare involvement, predominantly in child neglect cases [21,36,37]. One study that reviewed 639 child welfare cases in the US found that 79% of caregivers involved were misusing at least one substance [16]. Similarly, the 'Canadian Incidence Study of Reported Child Abuse and Neglect’ found that substance use was the most frequent root problem in caregiver-related cases [38]. These studies, along with our research, suggest a potential pathway between parental substance use, elevated risk for child maltreatment and subsequent out-of-home placements. This indicates that efforts to address parental substance use are needed. This may involve increasing access to evidence-based addiction treatments, parenting supports and other relevant social services.

Exposure to the child welfare system was also associated with physical abuse among our sample. This is supported by prior studies which indicate that youth in care are more likely than the general population to have experienced parental neglect, domestic violence, physical, and sexual abuse [1,3,7]. Given these findings, targeted supports to aid these youth cope with their traumatic experiences appears to be particularly warranted.

High school incompletion was another factor found to be associated with a history of being in government care. Low educational attainment among youth in government care is consistent with a wealth of research that indicates this population is much more likely to fail a grade, not graduate, have lower standardised test scores and lower post secondary education achievement levels [8,17,39-41]. When youth who have not completed high school are emancipated from government care, and do not have familial or financial support, the transition into financial independence can be unsuccessful and lead to instability. Research has found that low or a lack of educational attainment for youth in government care results in elevated rates of reliance on social assistance and many experiencing acute poverty [6,7,10]. Early interventions for youth currently in government care are needed to ensure a trajectory that follows and supports students through to high school completion in order to mitigate some of the poor outcomes identified.

Together these study findings highlight that youth with a history of being in government care are significantly more susceptible to substance use and street-involvement later in life, suggesting that action is needed on multiple fronts. For families on the cusp of having their children taken into government custody, parental support and training for parents has been associated with creating healthy families [15,42-44]. Given the association between parental substance use and youth exposure to the child welfare system, a focus on addiction treatment within parental support programs appears to be particularly relevant. For children and youth who are in government care and do not have a safe family environment to return to, evidence suggests that actions to identify permanent care options quickly and efficiently are advantageous [5,45-49]. Also needed are efforts to ensure that appropriate supports and services are in place after youth are emancipated from care, or 'age-out’ of the system. Independent living programs are initiatives that focus on life skills training for at-risk youth and involve assistance with setting up bank accounts, finding housing and employment, goal setting, developing healthy relationships, and conflict resolution. A small number of studies have found these programs to be associated with improved outcomes for youth in government care [5,6]; however, other studies have reported mixed results [50,51], indicating that more research is needed to ensure that newly emancipated youth are effectively supported by services and are able to better transition to independence.

This study has several limitations. First, as with all community-recruited research cohorts, the ARYS cohort is not a random sample and therefore may not generalise to other populations of street youth. Second, data collected was based on self report and thus could be subject to response biases, including socially desirable responding, which may have resulted in under reporting of illicit substance use and other stigmatised behaviours. As a result, the prevalence of some risk behaviours may have been underestimated in the present study. However, self reported risk behaviour has been shown to be largely accurate among adult substance-using populations [52] and also among various youth populations [53]. Furthermore, we acknowledge that there may be some unmeasured risk (e.g., assaults or traumatic events that occurred outside of time spent in child protection) or other confounding factors (e.g., length of time in care, multiple placements) that were not considered in our analyses. With respect to how we currently measure exposure to the child welfare system, the binary nature of participant response (yes vs. no) limits the interpretation of the findings. Specifically, important information concerning exposure to the child welfare system (e.g., number of placements, length of time in care) was not captured by our study instrument, including the various factors that have been shown to have a negative impact on long-term outcomes. For example, multiple care placements have been associated with negative impacts upon emotional and cognitive development, behavioural issues, and life-skills development [5,46,48,49,54,55]. Given that the intensity and frequency of exposure were not measured in this analysis, we recognise future analyses should seek to measure these important aspects of child welfare system exposure.

## Conclusions

Based on our study sample, street-involved youth in Vancouver are over 160 times more likely to have a history of being in government care compared to the general population of youth. Our study found that those with a history of being in government care were more likely to be of Aboriginal ancestry, have started using hard substances at an earlier age, have a history of physical abuse, have a parent that drank heavily or used illicit substances, and did not complete high school. Outcomes associated with the child welfare system have become a public health concern, and one that governments have failed to adequately address. However, these findings give policymakers potential areas for redress and demonstrate the need for interventions to support families and youth along the continuum of risk. This includes interventions to support at-risk families before government involvement is necessary, policies for children and youth currently in care, and services to help youth successfully transition out of care and into early adulthood.

## Competing interests

The authors declare that they have no competing interests.

## Authors’ contributions

The specific contributions of each author are as follows: BB, KD, TK and EW designed the study and wrote the protocol, BB managed the literature searches and BB and KD prepared the first draft of the analysis; PN conducted the statistical analyses with input from BB, KD; all authors contributed to the main content and provided critical comments on the final draft. All authors approved the final manuscript.

## Pre-publication history

The pre-publication history for this paper can be accessed here:

http://www.biomedcentral.com/1471-2458/14/197/prepub
